# Is there a link between all-cause mortality and economic fluctuations?

**DOI:** 10.1177/14034948211049979

**Published:** 2021-10-20

**Authors:** Iman Dadgar, Thor Norström

**Affiliations:** Swedish Institute for Social Research, Stockholm University, Stockholm, Sweden

**Keywords:** all-cause mortality, GDP, unemployment. Great Recession, error correction model

## Abstract

*Background:* All-cause mortality is a global indicator of the overall health of the population, and its relation to the macro economy is thus of vital interest. The main aim was to estimate the short-term and the long-term impact of macroeconomic change on all-cause mortality. Variations in the unemployment rate were used as indicator of temporary fluctuations in the economy. *Methods:* We used time-series data for 21 OECD countries spanning the period 1960–2018. We used four outcomes: total mortality (0+), infant mortality (<1), mortality in the age-group 20–64, and old-age mortality (65+). Data on GDP/capita were obtained from the Maddison Project. Unemployment data (% unemployed in the work force) were sourced from Eurostat. We applied error correction modelling to estimate the short-term and the long-term impact of macroeconomic change on all-cause mortality. *Results:* We found that increases in unemployment were statistically significantly associated with decreases in all mortality outcomes except old-age mortality. Increases in GDP were associated with significant lowering long-term effects on mortality. ***Conclusions:* Our findings, based on data from predominantly affluent countries, suggest that an increase in unemployment leads to a decrease in all-cause mortality. However, economic growth, as indicated by increased GDP, has a long-term protective health impact as indexed by lowered mortality.**

## Introduction

All-cause mortality is a classic indicator of the overall health of the population [1]. It is therefore of great concern to get a better understanding of the driving forces behind changes in mortality. Using cross-sectional time-series data for 21 countries, this work studied the potential role of macroeconomic fluctuations as indicated by changes in unemployment and per capita gross domestic product (GDP).

Intuition could easily make one believe that recessions can only be for the worse, but as described below the relation between economic fluctuations and population health is complex and seemingly contradictory. This may explain why the received wisdom concerning this relationship has undergone some quite substantial shifts. It is clear that economic downturns in past historical centuries led to severe malnutrition and starvation and thus worsened population health. Economic growth, on the other hand, was conducive to education, improved sanitation and living conditions, and, in the end, lowered mortality [2]. However, as demonstrated by Preston [3] there is a diminishing health return to economic growth, and there are even indications that economic downturns in highly industrialized societies may improve population health. The explanation to this counterintuitive finding is that although a downturn in all probability has a detrimental effect on some outcomes, such as mental health as indexed by suicide [4], this negative effect may be more or less offset by a beneficial impact on other dimensions of health. Several examples of such beneficial effects have been suggested and substantiated. A slow-down in the economy is thus associated with reduced overtime and work-related stress, less driving and fewer car crashes, less air pollution, and reduced intake of unhealthy products such as alcohol and tobacco [4-6]. Already in the early 20^th^ century there were reports (e.g. Ogburn and Thomas [7]) suggesting that economic booms were associated with increased mortality, while the opposite was true for economic downturns. However, these results were ignored for a long time, probably because they appeared to run counter to intuition. In the 1970s and 1980s, Harvey Brenner published a series of papers [8] suggesting marked negative influences of recessions on population health, as indexed by mortality rates. These findings attracted much interest; however, closer examinations of Brenner’s work [9] revealed serious methodological flaws, such as correlating trending time-series, and arbitrary specifications of lagged effects. The investigation by Ruhm [4] was one of the first well-designed studies in the field. On the basis of fixed-effects modelling of US state data for the period 1972–1991, his findings suggested that recessions are associated with improved health. More specifically, mortality from eight out of ten causes of death under study decreased during bad times, especially traffic fatalities. An important exception was suicide, which increased in downturns. Alluding to the title of his article, Ruhm ends with: ‘Are recessions good for your health? Surprisingly, the answer appears to be yes’. This finding was replicated by Tapia Granados’ study [10], based on US data for the period 1900–1996. Findings from some additional country studies [11, 12] point in the same direction. Some studies use cross-sectional time-series data including a large set of countries. Thus Gerdtham and Ruhm [6] analysed time-series data for 23 Organisation for Economic Co-operation and Development (OECD) countries for the period 1960–1997, and concluded that economic expansion was associated with increased all-cause mortality; these results were replicated in a similar study by Bilal et al. [13]. There are some studies that report non-significant associations between all-cause mortality and economic fluctuations, including one investigation based on Danish data [14], and another study relying on time-series data for 26 European Union countries [15]. Further, one study[16], based on Danish and US data, found that upturns in the economy were associated with decreases in mortality. In sum, although most of the well-designed studies on this field suggest a procyclical effect, the overall pattern of the findings is far from conclusive (see the review by Catalano et al. [5] for a similar conclusion). Moreover, previous research has generally focused on the immediate, contemporaneous health effect of the economy, typically gauged by oscillations of the unemployment rate. A large body of research suggests that unemployment is associated with a range of adverse health outcomes (see elsewhere for reviews [5, 17]). Perhaps the most succinct unemployment effect is noted on indicators of mental health [18]. For instance, studies at individual as well as aggregate level suggest a link between unemployment and suicide risk [18]. Other outcomes associated with unemployment status include heart disease mortality and all-cause mortality [17]. However, two studies based on Finish data [19, 20] found that the unemployment effect on mortality was weaker the higher the unemployment rate, which indicates the presence of health selection; that people with poor health run an elevated risk of becoming unemployed. To minimize this source of bias, Böckerman and Ilmakunnas [21] applied fixed-effects modelling of panel data pertaining to Finland. Their findings showed no association between unemployment and self-assessed health. As noted above, several studies have even reported negative relations between unemployment rates and various fatality rates.

However, the presence of health-protective long-term effects of economic growth seems quite plausible. A case in point is traffic fatalities. Research suggests that economic upturns are associated with increases in traffic deaths [4]; this is likely a short-term effect mainly due to the increased road traffic induced by an expanding economy. However, at least in high-income countries, it seems reasonable to expect a long-term effect of economic growth that is manifested in safer vehicles and roads that leads to improved traffic safety and reduced rates of traffic deaths. The hypothesis of such a protective long-term effect of GDP on traffic deaths was supported by a study based on panel data for 18 OECD countries [22]. A similar line of reasoning should be applicable to all-cause mortality. During the last half-century, the period we focus upon, there has been a marked and steady decrease in all-cause mortality in affluent countries (see below). The driving forces behind this development, as suggested in the literature [1, 3, 23, 24], include improvements in nutrition, housing, educational level and medical treatment. Because all of these factors are to varying degrees linked to economic growth, it seems reasonable to hypothesize a long-term beneficial impact of GDP on population health.

Another issue concerns the possible heterogeneity in the association between economic change and mortality, that is, that certain country characteristics may modify the association at issue. For instance, previous research suggests that the pernicious unemployment effect on suicide is weaker the more generous the unemployment protection of the country [15, 25]. In the present context it may be hypothesized that a possible beneficial association between economic growth and population health would be stronger the larger share of GDP that is spent on welfare provisions. To test this notion, the countries were sorted into three groups (low, medium and high; see Table I) based on their ranking on spending on public insurance systems (sources: OECD Social Expenditure Database and Gerdtham and Ruhm [6]). The main areas for social public spending include policies related to pensions, health, family and unemployment. In the low-country group the average spending on public insurance systems was 16.6 % of GDP during the period 1980–2018; the corresponding figures for the medium- and high-spending groups were 20.3 and 24.5 %, respectively.

**Table I.** Descriptive statistics (period average) for all-cause mortality (number of deaths per 100,000), unemployment (% of the work force), and GDP/capita ($1,000). Country-group signifies degree of public spending on social insurance systems as % of GDP where 1=low, 2=medium and 3=high public spending.

Country	Mortality				Unemployment	GDP	Country-group	Observation period
	Infant	20-64	65+	Total				
Australia	978.6	303.7	4823.1	601.0	5.5	32.1	1	1960–2018
Austria	1283.1	328.6	5371.6	666.4	3.3	28.3	3	1960–2018
Belgium	1154.8	332.9	5398.2	667.5	7.4	27.1	2	1960–2016
Canada	1232.3	306.8	4574.9	633.1	7.4	31.0	1	1960–2005
Denmark	854.5	320.5	5209.0	637.5	5.5	32.8	3	1960–2018
Finland	744.3	364.9	5526.5	688.2	6.5	25.5	3	1960–2018
France	956.8	337.8	4578.7	599.4	6.8	27.3	3	1960–2014
Germany	1162.9	322.3	5361.3	658.6	5.2	31.6	3	1960–2018
Greece	1470.6	254.2	4872.0	584.7	10.3	18.5	1	1974–2017
Ireland	1166.2	337.2	6056.1	723.9	9.1	29.1	1	1960–2015
Italy	1467.3	282.7	4935.3	606.3	8.7	27.0	2	1960–2017
Japan	791.5	265.5	4516.2	549.9	2.8	25.4	1	1960–2018
New Zealand	1105.3	329.2	5165.0	649.0	3.9	24.3	2	1960–2016
Norway	795.9	266.4	4841.7	576.5	2.9	56.2	3	1960–2016
Portugal	2505.2	352.3	5783.8	748.4	7.3	17.3	1	1974–2018
Spain	1193.2	282.8	4790.5	590.1	15.1	21.3	2	1972–2017
Sweden	672.3	250.9	4730.4	553.8	4.6	29.9	3	1960–2018
Switzerland	887.2	265.0	4638.9	561.3	2.0	47.0	2	1960–2017
The Netherlands	801.5	264.6	4886.9	578.3	4.9	31.5	3	1960–2018
United Kingdom	1077.8	319.7	5406.7	657.8	5.8	25.5	2	1960–2016
United States of America	1364.5	395.9	5065.1	694.4	6.0	36.4	1	1960–2007

## Study aims

The main aim of this paper is thus to assess the short-term as well as the long-term impact of macroeconomic change on all-cause mortality, using data for 21 OECD countries spanning the period 1960–2018. In keeping with most previous studies, we will use changes in the unemployment rate as indicator of temporary fluctuations in the economy. The possible long-term impact of economic growth on mortality will be assessed by error correction modelling of the effect of GDP.

In sum, the main potential contributions of our paper are (i) that we span a long time period and include a large set of countries, making the findings more generalizable than those from previous studies; and (ii) that we assess the short-term as well as the long-term effect of economic change on mortality – this is an important issue because the short-term and the long-term effect may have opposite signs, a phenomenon that has not been addressed in previous research.

## Data

The study comprises 21 OECD countries, and the longest observation period is 1960–2018, though it is somewhat shorter for some countries (see Table I). Age-specific mortality data were obtained from the World Health Organization (WHO) Mortality Data Base (Geneva). We used four outcomes: total mortality (0+), infant mortality (<1), working-age mortality (20–64), and old-age mortality (65+). The outcomes were expressed as number of deaths per 100,000 population, and were age-standardized following WHO World Standard. Unemployment data (% unemployed in the work force) were sourced from Eurostat. Data on gross domestic product/capita (GDP), expressed in Purchasing Power Parity (PPP), converted into US dollars of 1990 years value, were obtained from the Maddison Project [26].

## Statistical analysis

Our analytical strategy for estimating the relation between mortality and the two economic indicators is to apply error correction modelling (ECM), which is a feasible approach when short- and long-term dynamics are addressed [27]. Although ECM is a standard modelling tool in economics, it is, as pointed out by De Boef and Keele [27], under-utilized in other branches of social science.

However, prior to performing ECM it is necessary to carry out some initial analyses with respect to the variables where a long-term effect may be expected, that is, GDP and mortality. These analyses comprised two steps; first, we tested for unit root using the Fisher-Type ADF panel unit root test [28]. If the independent and dependent variables prove to be integrated of the order *I*(1), the next step is to test whether they are cointegrated. Two variables, *X* and *Y*, are cointegrated if there exists a linear combination of *X* and *Y* that is stationary around which the two series fluctuate. This implies that if *X* drifts off, *Y* is bound to follow suit, and in the long run the series will not diverge far apart. The theory of cointegration stems from Engle and Granger [29], and empirical examples include the relation between GDP and traffic fatalities [22]. We used the panel cointegration tests developed by Westerlund [30], denoted P_t_ and P_a_. Simulation results [30] indicate that the tests have better small-sample properties and power than other commonly used panel cointegration tests. The simulations further indicate that each of the two tests has its own merits and limitations, and should thus be considered jointly. The tests accommodate various forms of heterogeneity, and also generate *p*-values that are robust against cross-sectional dependencies via bootstrapping [30]. As detailed below, the outcome of these initial analyses suggested that both GDP and mortality were integrated of the order *I*(1), and that they were cointegrated according to at least of one of the two tests; the conditions for performing ECM were thus fulfilled.

Following standard specifications [27], our error correction model was specified as follows:

(2) $$\begin{array}{c} \Delta LnMortality_{it}=\alpha +\beta_{0}\Delta GDP_{it}\\ +\beta_{1}LnMortality_{it-1}+\beta_{2}GDP_{it-1}\\ +\beta_{3}\Delta Unemployment_{it}+\beta_{i}CD_{i}+\varepsilon_{it} \end{array}$$

Following common practice (e.g. Ruhm [4]), we used the natural log of mortality as outcome. In this equation, *β*_0_ indicates the instantaneous, short-term effect of a change in GDP on mortality, while *β*_1_ estimates the speed at which the long-term effect operates. If such an effect actually exists, the estimate of *β*_1_ should be negative and statistically significant. The model assumes that the long-term effect decays geometrically. The total long-term effect is calculated as -*β*_2/_*β*_1_. *CD* is a vector of country dummies.

A complication with cross-sectional time-series data is the likely presence of serial and spatial (cross-country) dependence of the errors, which yields a downward bias of the estimated standard errors (SEs). As a remedy, we applied a modelling technique that addresses this complication as follows. First, it accounts for spatial dependence of the errors by applying the more conservative panel-corrected SEs suggested by Beck and Katz [31]. Simulation results indicated that the panel-corrected SEs performed excellently; the procedure also yields a correction for any panel heteroscedasticity [31]. Secondly, our modelling technique accounts for temporal dependence by including panel-specific autoregressive parameters for estimation of residual autocorrelation. In addition, we included country-specific dummies to account for possible country-specific heterogeneity. It should be emphasized that our analytical design implies that only temporal within-country variation is exploited.

The analyses reported in the main tables were carried out on unweighted data. However, as a sensitivity test, the main analyses were also performed on data where the observations were weighted by the square root of the country population. To test for possible gender-specific effects, we estimated separate models for females and males. We also estimated separate models for the three country-groups with different levels of spending on public insurance systems.

We used the Bewley transformation regression [32] to estimate SEs and significance levels of the long-term effect in the ECMs. All statistical analyses were performed with Stata, V.15 (StataCorp).

## Results

**Figure 1.**
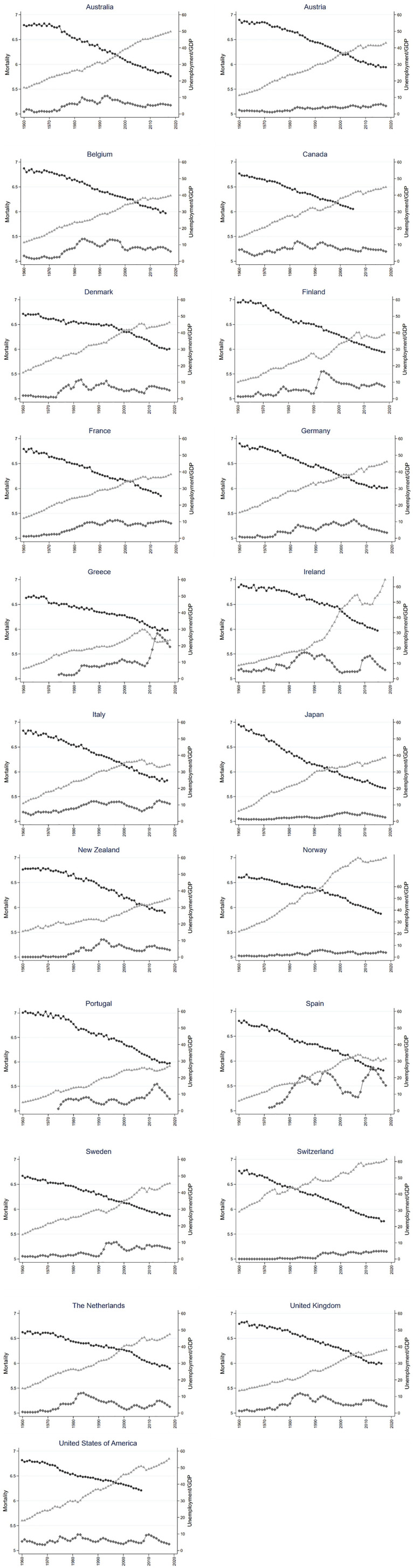
Trends in all-cause mortality per 100,000 in log (black circle), unemployment rate (diamond) and GDP per capita (US$1000, triangle).

Table I displays descriptive statistics. As appears in Figure 1, there was a steady growth in GDP in all countries during the study period. Another trait common to all countries is the decreasing trend in mortality. In contrast, the trajectories in unemployment do not display any common pattern; for most countries there is no marked trend, but rather irregularly occurring peaks and troughs.

The results of the panel unit root tests of GDP and various mortality rates (Table II, panel A) suggest that for all variables the null hypothesis of unit root cannot be rejected by any of the four statistics. Given this outcome, we proceed to test whether the relation between GDP and mortality is cointegrated. Table II (panel B) shows that the null hypothesis of no cointegration was rejected by at least one of the two panel tests in all age groups. We thus proceeded to estimate the error correction models. The outcome is shown in Table III. The percentage change in mortality from a one-unit increase in an explanatory variable, *X*, is obtained by the expression, $\left(exp\left[\hat{\beta }\right]-1\right)*100$ , where $\hat{\beta }$ is the estimated effect of *X.* An increase in the unemployment rate by 1 percentage point was thus estimated to give a decrease in total mortality by 0.3%. The corresponding figure for infant mortality and mortality in the age-group 20–64 years were 0.8 and 0.3%, respectively, while the estimate for old-age mortality was not statistically significant. A one-unit increase in GDP (in $1000, which on average corresponds to a relative increase in GDP by 3.3%) had no statistically significant instantaneous effect on infant mortality or old-age mortality, while the significant estimates for 20–64 years mortality and total mortality imply a reduction in mortality by 0.3 and 0.4%, respectively. Now turning to the long-term effect of a one-unit increase in GDP (i.e. corresponding to an increase by 3.3%), the results suggest a reduction in total mortality by 3.8%; the corresponding figures for infant mortality, 20–64 years mortality and old-age mortality were 4.6, 7.0 and 3.1%, respectively (all these estimates were statistically significant).

**Table II.** Unit root tests (Panel A), and cointegration tests (Panel B). Panel A. Fisher-Type ADF panel unit root tests of H0: All panels contain unit roots against H1: At least one panel is stationary.

Test		GDP/capita		Unemployment		Infant mortality		20–64		Old-age mortality		Total	
		Statistic	*p*	Statistic	*p*	Statistic	*p*	Statistic	*p*	Statistic	*p*	Statistic	P
Inverse chi–squared(36)	P	34.26	0.80	27.99	0.95	54.14	0.10	5.96	>0.99	2.23	>0.99	2.60	>0.99
Inverse normal	Z	2.33	0.99	0.65	0.74	−0.44	0.33	10.51	>0.99	8.23	>0.99	9.19	>0.99
Inverse logit t(94)	L	2.49	>0.99	0.60	0.73	−0.86	0.20	12.35	>0.99	8.47	>0.99	9.89	>0.99
Modified inv. chi–squared	pm	−0.84	0.80	−1.53	0.94	1.32	0.09	−3.93	>0.99	−4.34	>0.99	−4.30	>0.99

**Table:** Panel B. Westerlund panel cointegration tests of H0: no cointegration for panels against H1: cointegration for all panels.

	Infant mortality			20–64			Old-age mortality			Total mortality		
	Statistic	*p*	Robust P	Statistic	*p*	Robust P	Statistic	*p*	Robust P	Statistic	*p*	Robust P
Pa	−13.04	0.001	<0.001	−0.58	0.759	0.620	−13.51	<0.001	<0.001	−14.40	<0.001	<0.001
Pt	−11.55	0.016	<0.001	−6.88	<0.001	<0.001	−14.73	<0.001	<0.001	−13.44	<0.001	<0.001

**Table III.** Estimates of GDP/capita ($1,000) and unemployment on all-cause mortality rates (per 100,000) based on error correction models (ECM). Models include country dummies. Panel-corrected standard errors accounting for spatial dependence and panel heteroscedasticity.

Age-group	N	ΔGDP_t_			ΔUnemployment_t_			Mortality_t−1_			GDP_t-1_			Long-term effect of GDP		
		Est	SE	*p*	Est	SE	*p*	Est	SE	*p*	Est	SE	*p*	Est	SE	*p*
Infant	1135	−0.0018	0.0040	0.6629	−0.0079	0.0027	0.0034	−0.0404	0.0070	<0.001	−0.0019	0.0005	<0.001	−0.0466	0.0003	<0.001
20–64	1155	−0.0035	0.0011	0.0018	−0.0031	0.0008	<0.001	−0.0103	0.0049	0.0342	−0.0007	0.0001	<0.001	−0.0724	0.0003	<0.001
65+	1146	−0.0043	0.0023	0.0641	−0.0025	0.0014	0.0659	−0.0396	0.0098	<0.001	−0.0012	0.0002	<0.001	−0.0312	0.0002	<0.001
Total	1135	−0.0042	0.0018	0.0219	−0.0030	0.0011	0.0053	−0.0244	0.0073	<0.001	−0.0009	0.0002	<0.001	−0.0388	0.0002	<0.001

To put the key estimates into perspective, and to facilitate comparisons among them, we converted them into elasticities, confining ourselves to the effects on mortality in the whole population (Total). The outcome suggests that the elasticity for the short-term effect in GDP is −0.1259; i.e. an increase in GDP by 1% would yield an instantaneous decrease in total mortality of 0.1259%. The corresponding figure for the long-term effect of GDP is −1.1429, and for unemployment −0.0181.

The estimates from the analyses based on weighted data (Table SI) differ little from these based on unweighted data. With regard to gender differences, the only more systematic pattern is that the long-term effect of GDP tends to be somewhat stronger in males than in females (Table SII). Finally, public spending on social insurance systems does not seem to modify the response of mortality to macroeconomic changes; the effect estimates display no systematic differences across the three country-groups (Table SIII).

## Discussion

Economic upturns seem to have beneficial effects on some causes of death, and detrimental on others; to assess the net effect of macroeconomic change it is thus feasible to focus upon a global outcome, such as all-cause mortality. In this study we used panel data for 21 OECD countries, spanning the period 1960–2018 to estimate the association between all-cause mortality and two key macroeconomic indicators, GDP and unemployment. The aim was to assess not only the short-term health effect of temporary fluctuations in the economy, as indicated by fluctuations in unemployment, but in addition to estimate the long-term effect of economic growth in GDP. We found that an increase in unemployment is associated with an improvement in population health (as indexed by total mortality). The size of the estimated effect, 0.3% decrease in mortality following a 1 percentage point increase in unemployment, is somewhat lower than most of the previously reported estimates, ranging from 0.4% [4] over 1.1% [33] to 2.2% [10]. It may seem surprising that also infant mortality was found to be negatively related to unemployment. However, a couple of previous studies that have reported similar findings substantiate some plausible underlying mechanisms, viz. that recessions tend to lower levels of air pollution [34], and to generate improved health behaviour in mothers (e.g. less smoking and drinking) [35].

In contrast, our findings suggested a short-term as well as a long-term protective effect of growth in GDP, where the long-term effect was markedly stronger than the short-term impact. To our knowledge, there is no other study focusing on all-cause mortality that has elucidated this issue, implying that we lack a basis for comparisons. However, one study that applied the same analytical strategy (ECM), but a more narrow outcome (traffic fatalities), also reported a long-term beneficial impact of GDP [22].

An important issue is, of course, what implications our findings have with regard to future research and policy measures. The health-economic research that has emerged in the vein of Ruhm’s influential work (e.g. [4]) makes it tempting to conclude that ‘the pro-cyclical character of mortality fluctuations is beginning to be a proven fact’ [36]. Our finding of a marked protective long-term effect of economic growth makes such a conclusion disputable. However, notwithstanding any beneficial long-term effect of economic growth, our findings do suggest that temporary economic upturns, as indicated by decreased unemployment, tend to have deleterious effects on population health. An obvious task is to identify the mechanisms underlying these effects. As noted above in the Introduction, several mechanisms have been suggested and at least partly corroborated, including increased work-related stress, more road traffic and car crashes, higher levels of air pollution, and increased consumption of unhealthy products such as alcohol and tobacco. To make some progress in this area, it seems urgent to investigate the possible presence of socio-cultural contingencies; that is, is the relation between economic fluctuations and these mechanisms modified by social and cultural characteristics of the country? A better understanding of these relations and their socio-cultural contingencies would potentially enable the tailoring of policy measures to mitigate these adverse health effects of economic upturns.

Before concluding, we will note the major strengths and limitations of the study. Our data comprise a large number of countries, and cover a fairly long time period. However, these data are confined to affluent countries during a prosperous historical epoch, which of course limits the generalizability of our findings. Our estimates rely on within-country variation only, thus avoiding the potential bias that likely arises from cross-country co-variation. However, the risk of omitted variable bias cannot be dismissed in the present kind of research; i.e. that the findings have been distorted by the omission of some factor that is related to mortality as well as to the macroeconomic indicators. We applied a modelling approach (ECM) that is novel to the field, and which yielded new insights into the dynamics of the relation between mortality and macroeconomic change. However, the uniqueness of the findings regarding the long-term effect of GDP implies that we have little external evidence to validate them against, so these findings should be probed further in future research.

Bearing the above caveats in mind, we conclude that our findings suggest that an increase in unemployment yields an instantaneous decrease in all-cause mortality among infants and in the working-age population. Further, we found a protective short-term as well as long-term effect of GDP.

## Key points

- All-cause mortality is a global indicator of the overall health of the population, and its relation to the macro economy is thus of vital interest.
- On the basis of time-series data for 21 OECD countries spanning the period 1960–2016, we found that increases in unemployment had a statistically significant association with decreases in mortality.
- Economic growth, as indicated by increased GDP, had a long-term protective health impact as indexed by lowered mortality.

## Supplemental Material

sj-docx-1-sjp-10.1177_14034948211049979 – Supplemental material for Is there a link between all-cause mortality and economic fluctuations? Click here for additional data file. Supplemental material, sj-docx-1-sjp-10.1177_14034948211049979 for Is there a link between all-cause mortality and economic fluctuations? by Iman Dadgar and Thor Norström in Scandinavian Journal of Public Health
