# Supplementary material for: Is there a link between all-cause mortality and economic fluctuations?
Source: Scand J Public Health. 2021 Oct 20;50(1):6–15. doi: 10.1177/14034948211049979 (PMC8808227; doi:10.1177/14034948211049979)
Supplement: sj-docx-1-sjp-10.1177_14034948211049979 – Supplemental material for Is there a link between all-cause mortality and economic fluctuations? [file sj-docx-1-sjp-10.1177_14034948211049979.docx]

| Table S1: Estimates of GDP/capita ($1,000) and unemployment on all-cause mortality rates (per 100 000) based on error correction models (ECM). Models include country dummies. Observations weighted by the square root of the country population. Panel-corrected standard errors accounting for spatial dependence and panel heteroscedasticity. | | | | | | | | | | | | | | | | | |
| --- | --- | --- | --- | --- | --- | --- | --- | --- | --- | --- | --- | --- | --- | --- | --- | --- | --- |
|  |  | ΔGDP_t_ | | | ΔUnemployment_t_ | | | Mortality_t−1_ | | | GDP_t-1_ | | | Long-term effect of GDP | | | |
| Age-group | N | Est | SE | p | Est | SE | p | Est | SE | p | Est | SE | p | Est | SE | p |  |
| Infant | 1135 | -0.0016 | 0.0035 | 0.6430 | -0.0070 | 0.0027 | 0.0083 | -0.0346 | 0.0065 | <0.001 | -0.0015 | 0.0005 | 0.0028 | -0.0436 | 0.0003 | <0.001 |  |
| 20-64 | 1155 | -0.0041 | 0.0014 | 0.0024 | -0.0038 | 0.0008 | <0.001 | -0.0101 | 0.0054 | 0.0633 | -0.0006 | 0.0002 | <0.001 | -0.0573 | 0.0002 | <0.001 |  |
| 65+ | 1146 | -0.0035 | 0.0026 | 0.1701 | -0.0029 | 0.0014 | 0.0371 | -0.0499 | 0.0109 | <0.001 | -0.0015 | 0.0003 | <0.001 | -0.0311 | 0.0002 | <0.001 |  |
| Total | 1135 | -0.0039 | 0.0021 | 0.0630 | -0.0035 | 0.0011 | 0.0020 | -0.0330 | 0.0084 | <0.001 | -0.0012 | 0.0002 | <0.001 | -0.0354 | 0.0002 | <0.001 |  |
|  |  |  |  |  |  |  |  |  |  |  |  |  |  |  |  |  |  |

| Table S2: Estimates of GDP/capita ($1,000) and unemployment on all-cause mortality rates (per 100 000) based on error correction models (ECM). Models include country dummies. Models are estimated separately for females and males. Panel-corrected standard errors accounting for spatial dependence and panel heteroscedasticity. | | | | | | | | | | | | | | | | | | |
| --- | --- | --- | --- | --- | --- | --- | --- | --- | --- | --- | --- | --- | --- | --- | --- | --- | --- | --- |
|  |  |  | ΔGDP_t_ | | | ΔUnemployment_t_ | | | Mortality_t−1_ | | | GDP_t-1_ | | | Long-term effect of GDP | | | |
| Age-group | Gender | N | Est | SE | p | Est | SE | p | Est | SE | p | Est | SE | p | Est | SE | p |  |
| Infant | Males | 1135 | -0.0039 | 0.0051 | 0.4498 | -0.0088 | 0.0032 | 0.0067 | -0.0517 | 0.0095 | <0.001 | -0.0027 | 0.0008 | <0.001 | -0.0518 | 0.0004 | <0.001 |  |
|  | Females | 1135 | 0.0021 | 0.0055 | 0.6973 | -0.0054 | 0.0036 | 0.1300 | -0.0610 | 0.0095 | <0.001 | -0.0030 | 0.0007 | <0.001 | -0.0487 | 0.0004 | <0.001 |  |
| 20-64 | Males | 1155 | -0.0037 | 0.0013 | 0.0049 | -0.0028 | 0.0009 | 0.0016 | -0.0118 | 0.0056 | 0.0351 | -0.0010 | 0.0002 | <0.001 | -0.0847 | 0.0003 | <0.001 |  |
|  | Females | 1155 | -0.0031 | 0.0014 | 0.0261 | -0.0032 | 0.0009 | <0.001 | -0.0231 | 0.0058 | <0.001 | -0.0007 | 0.0001 | <0.001 | -0.0296 | 0.0001 | <0.001 |  |
| 65+ | Males | 1146 | -0.0042 | 0.0023 | 0.0643 | -0.0017 | 0.0013 | 0.2010 | -0.0355 | 0.0101 | <0.001 | -0.0014 | 0.0002 | <0.001 | -0.0407 | 0.0003 | <0.001 |  |
|  | Females | 1146 | -0.0044 | 0.0025 | 0.0751 | -0.0033 | 0.0015 | 0.0256 | -0.0376 | 0.0093 | <0.001 | -0.0010 | 0.0002 | <0.001 | -0.0266 | 0.0002 | <0.001 |  |
| Total | Males | 1135 | -0.0043 | 0.0018 | 0.0189 | -0.0025 | 0.0011 | 0.0217 | -0.0211 | 0.0074 | 0.0044 | -0.0012 | 0.0002 | <0.001 | -0.0545 | 0.0003 | <0.001 |  |
|  | Females | 1135 | -0.0042 | 0.0020 | 0.0396 | -0.0036 | 0.0012 | 0.0028 | -0.0283 | 0.0074 | <0.001 | -0.0008 | 0.0002 | <0.001 | -0.0279 | 0.0002 | <0.001 |  |

Table S3: Estimates of GDP/capita ($1,000) and unemployment on all-cause mortality rates (per 100 000) based on error correction models (ECM). Models include country dummies. Models are estimated separately for country-groups with varying degrees of social spending on social insurance systems as % of GDP where 1=low, 2=medium and 3=high public spending.. Panel-corrected standard errors accounting for spatial dependence and panel heteroscedasticity.

|  |  |  | ΔGDP_t_ | | | ΔUnemployment_t_ | | | Mortality_t−1_ | | | GDP_t-1_ | | | Long-term effect of GDP | | |
| --- | --- | --- | --- | --- | --- | --- | --- | --- | --- | --- | --- | --- | --- | --- | --- | --- | --- |
| Country-group | Age-group | N | Est | SE | p | Est | SE | p | Est | SE | p | Est | SE | p | Est | SE | p |
| 1 | Infant | 350 | -0.0099 | 0.0062 | 0.1127 | -0.0127 | 0.0042 | 0.0025 | -0.0458 | 0.0110 | <0.001 | -0.0019 | 0.0008 | 0.0118 | -0.0423 | 0.0004 | <0.001 |
|  | 20-64 | 370 | -0.0048 | 0.0020 | 0.0154 | -0.0027 | 0.0013 | 0.0420 | -0.0173 | 0.0087 | 0.0477 | -0.0006 | 0.0003 | 0.0203 | -0.0353 | 0.0001 | <0.001 |
|  | 65+ | 361 | 0.0006 | 0.0030 | 0.8510 | -0.0017 | 0.0020 | 0.3998 | -0.0646 | 0.0137 | <0.001 | -0.0019 | 0.0004 | <0.001 | -0.0293 | 0.0002 | <0.001 |
|  | Total | 350 | -0.0014 | 0.0026 | 0.5943 | -0.0023 | 0.0017 | 0.1570 | -0.0468 | 0.0112 | <0.001 | -0.0015 | 0.0003 | <0.001 | -0.0316 | 0.0002 | <0.001 |
| 2 | Infant | 327 | 0.0066 | 0.0064 | 0.3021 | -0.0008 | 0.0041 | 0.8366 | -0.0520 | 0.0168 | 0.0022 | -0.0034 | 0.0014 | 0.0185 | -0.0655 | 0.0005 | <0.001 |
|  | 20-64 | 327 | -0.0026 | 0.0020 | 0.1950 | -0.0037 | 0.0011 | <0.001 | -0.0238 | 0.0119 | 0.0466 | -0.0016 | 0.0004 | <0.001 | -0.0656 | 0.0004 | <0.001 |
|  | 65+ | 327 | -0.0045 | 0.0033 | 0.1746 | -0.0029 | 0.0019 | 0.1269 | -0.1191 | 0.0294 | <0.001 | -0.0043 | 0.0010 | <0.001 | -0.0362 | 0.0004 | <0.001 |
|  | Total | 327 | -0.0040 | 0.0027 | 0.1348 | -0.0034 | 0.0015 | 0.0241 | -0.0684 | 0.0223 | 0.0024 | -0.0029 | 0.0008 | <0.001 | -0.0424 | 0.0003 | <0.001 |
| 3 | Infant | 458 | -0.0080 | 0.0069 | 0.2409 | -0.0148 | 0.0051 | 0.0039 | -0.0541 | 0.0136 | <0.001 | -0.0029 | 0.0010 | 0.0024 | -0.0537 | 0.0004 | <0.001 |
|  | 20-64 | 458 | -0.0039 | 0.0019 | 0.0392 | -0.0035 | 0.0015 | 0.0191 | -0.0195 | 0.0090 | 0.0316 | -0.0012 | 0.0002 | <0.001 | -0.0596 | 0.0004 | <0.001 |
|  | 65+ | 458 | -0.0067 | 0.0034 | 0.0513 | -0.0011 | 0.0024 | 0.6289 | -0.0432 | 0.0158 | 0.0064 | -0.0012 | 0.0003 | <0.001 | -0.0285 | 0.0003 | <0.001 |
|  | Total | 458 | -0.0059 | 0.0027 | 0.0267 | -0.0023 | 0.0019 | 0.2094 | -0.0280 | 0.0115 | 0.0158 | -0.0010 | 0.0003 | <0.001 | -0.0367 | 0.0003 | <0.001 |
